# Supplementary material for: Properties of a Single Amino Acid Residue in the Third Transmembrane Domain Determine the Kinetics of Ambient Light-Sensitive Channelrhodopsin
Source: Int J Mol Sci. 2023 Mar 6;24(5):5054. doi: 10.3390/ijms24055054 (PMC10003734; doi:10.3390/ijms24055054)
Supplement: Supplementary file 1 [file ijms-24-05054-s001.zip › ijms-2238858-supplementary.pdf]

Supplementary materials

a

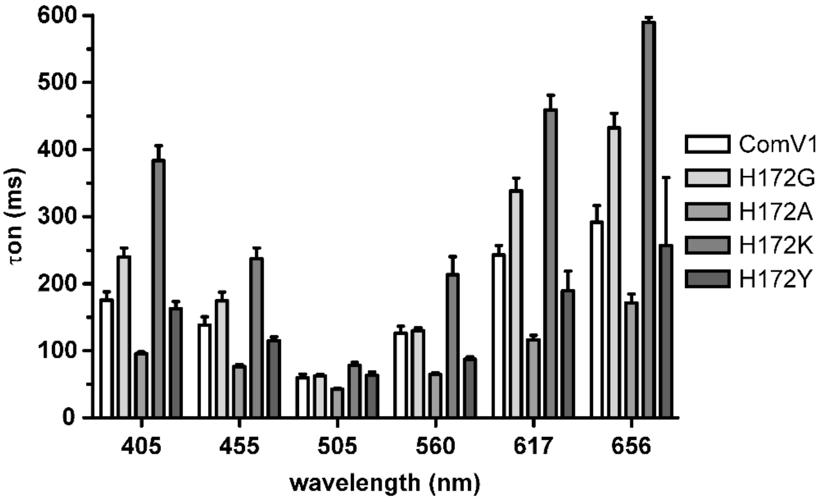

b

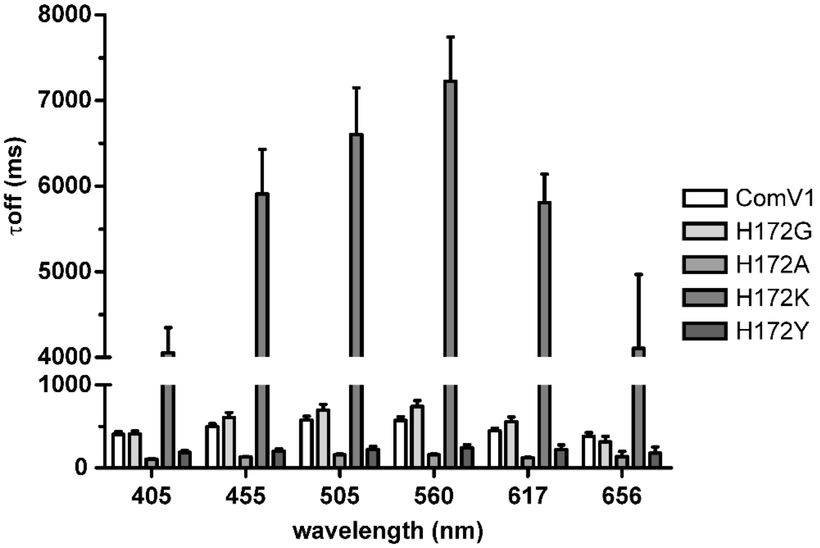

Figure S1 Comparison between ComV1 and its mutants

Comparison of τon (a) and τoff (b) between ComV1 and its mutants. The data are shown as mean ± SEM (ComV1 n = 18–19, H172G n = 8, H172A n = 8, H172K n = 8, H172Y n = 8). The data were analyzed using Dunnett's multiple comparison test; they are presented in Table S1 (*p < 0.05, **p < 0.01).

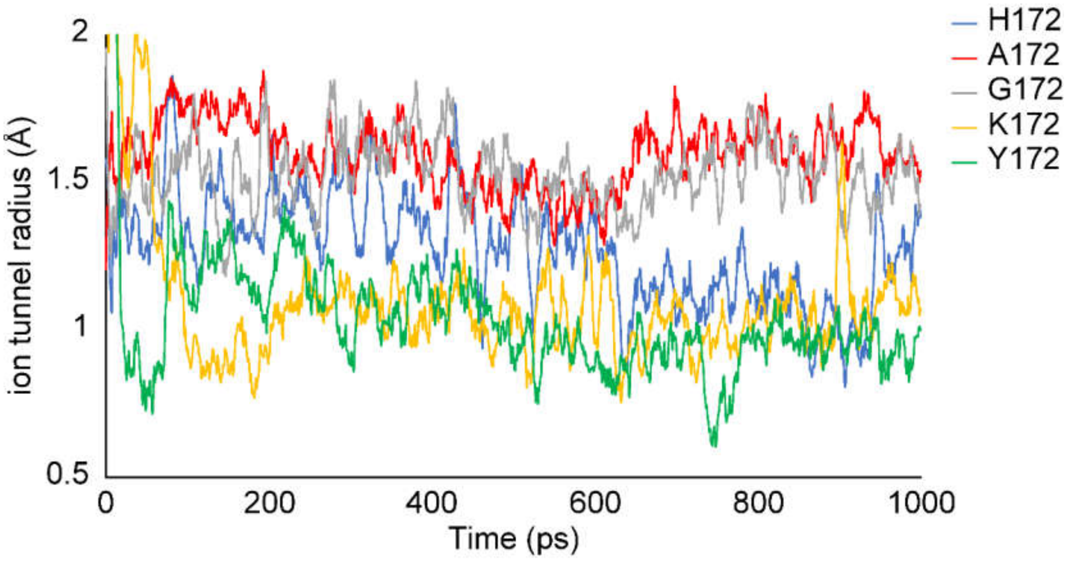

Figure S2 Structural analysis with MD simulation

Comparison of ion tunnel radius in the ground state between ComV1 and its mutants. The first structural model was built with homology modelling using the structure of PDBID: 4YZI as the template.

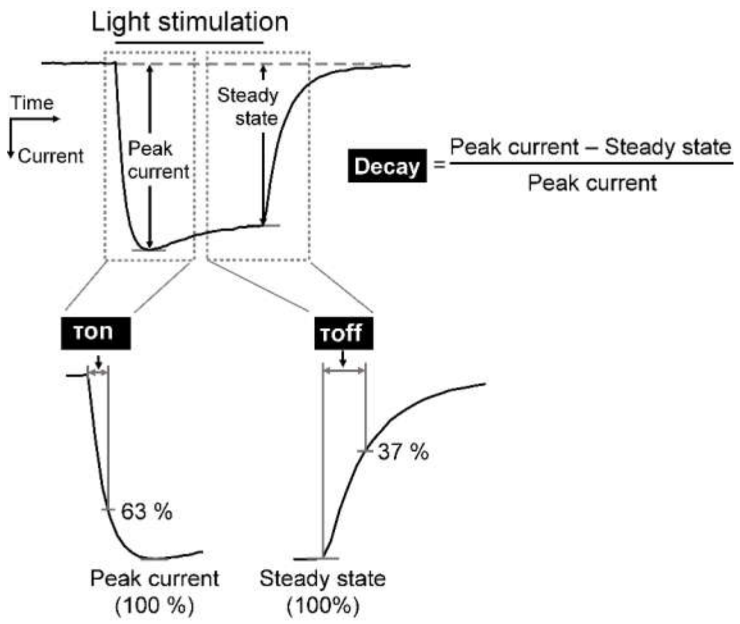

Figure S3 Graphical description of τon, τoff, and decay

τon is the time for the photocurrent to reach 63% of the peak current and τoff is the time for the photocurrent to reduce to 37% of the steady-state current. Decay is the value obtained by dividing the difference between the peak current and steady-state current by the peak current.

Table S1 Statistical analysis of photocurrent of each channelrhodopsin stimulated by light of various wavelengths in Fig. 2d.

One-way ANOVA with Dunnett's multiple comparison test vs. ComV1 (*p<0.05, **p<0.01).

Photocurrent	p-value summary					
	405 nm	455 nm	505 nm	560 nm	617 nm	656 nm
H172G						
H172A	**	**	**	*	**	*
H172K			**	*		**
H172Y	**	**	**	**	**	**

Table S2 Statistical analysis of τon of each channelrhodopsin stimulated by light of various wavelengths in Fig. S2a.

One-way ANOVA with Dunnett's multiple comparison test vs. ComV1 (*p<0.05, **p<0.01).

τon	p-value summary					
	405 nm	455 nm	505 nm	560 nm	617 nm	656 nm
H172G	**				**	
H172A	**	**	*	**	**	
H172K	**	**	*	**	**	**
H172Y						

Table S3 Statistical analysis of τoff of each channelrhodopsin stimulated by light of various wavelengths in Fig. S2b.

One-way ANOVA with Dunnett's multiple comparison test vs. ComV1 (*p<0.05, **p<0.01).

τoff	p-value summary					
	405 nm	455 nm	505 nm	560 nm	617 nm	656 nm
H172G						
H172A						
H172K	**	**	**	**	**	**
H172Y						

Table S4 The sequences of primers used PCR for producing mutants

	Forward	Reverse
H172R	CGCCTGAGCAACCTGACCGGCCTGAA	GATCAGGATCACAGGACAGGTCAGCA
H172G	GGACTGAGCAACCTGACCGGCCTGAA	GATCAGGATCACAGGACAGGTCAGCA
H172A	GCCCTGAGCAACCTGACCGGCCTGAA	GATCAGGATCACAGGACAGGTCAGCA
H172K	AAACTGAGCAACCTGACCGGCCTGAA	GATCAGGATCACAGGACAGGTCAGCA
H172Y	TACCTGAGCAACCTGACCGGCCTGAA	GATCAGGATCACAGGACAGGTCAGCA
